# Burden of comorbid disease, treatment, and healthcare utilization among people with HIV: A retrospective, cross-sectional study from a large healthcare organization

**DOI:** 10.1017/S0950268826101265

**Published:** 2026-03-24

**Authors:** Clara Weil, Sivan Gazit, Arlene Nugent, Liza Murrison, Moshe Hoshen, Ana Pires dos Santos, Itzchak Levy

**Affiliations:** 1 Maccabi Healthcare Services, Israel; 2 AbbVie Inc, USA; 3 Infectious Diseases Unit, Sheba Medical Center, Israel; 4 Sackler School of Medicine, Tel Aviv University, Israel

**Keywords:** ART use pattern, clinical burden, comorbidities, gaps in care, healthcare utilization, HIV, hospitalizations, Maccabi healthcare services, real-world evidence

## Abstract

This retrospective, cross-sectional study assessed the burden of HIV in Israel’s Maccabi Healthcare Services in 2022. Among 2.6 million individuals assessed (lookback period: > 2 decades), 1973 PWH were identified and age-sex-matched (1:5) to 9,865 randomly selected controls without HIV. We compared sociodemographic, clinical characteristics, and healthcare resource utilization (HRU; Jan–Dec 2022) between people with and without HIV and characterized antiretroviral therapy (ART) treatment patterns among PWH (Jan–Dec 2022). Compared to controls, PWH had a higher lifetime prevalence (since 1988; *P* < 0.001) of several comorbid conditions, including liver disease, chronic kidney disease, any cancer, and hepatitis B and C infections. Additionally, PWH had a higher rate of anxiety and depression (26.2% vs. 13.5%; *P* < 0.001). PWH showed higher annual HRU than controls, including ~2-fold higher hospitalizations (≥1 new admissions; *P* < 0.001) and frequent use of emergency, urgent, primary, specialist, and nursing care (P < 0.05). Among 1907 PWH with ≥1 ART prescription, 78.1% had ≥90% coverage in 2022, although 69.1% experienced ART interruption and 7.2% discontinuation, the latter associated with mental health issues. This study recognizes critical gaps in care that could inform strategies to improve clinical outcomes and resource allocation in health systems for PWH.

## Introduction

Contemporary antiretroviral therapy (ART) has transformed HIV-1 infection into a chronic, manageable illness. However, even with effective ART, people with HIV (PWH) experience substantial clinical burden due to chronic inflammation, long-term drug toxicity, and elevated multimorbidity that increases with age, including cardiovascular disease, diabetes, chronic kidney disease, malignant neoplasms, and mental health conditions [1, 2]. In addition, lifelong ART introduces multiple psychosocial challenges for PWH, including HIV-related stigma, reminders of their diagnosis from daily medication, anxiety about possible unintended disclosure of HIV status, and the ongoing need for adherence [3]. The cumulative impact of these burdens translates into higher healthcare costs due to hospitalizations, laboratory tests, ART and non-ART medications, and a greater need for specialist, primary, and nursing care [4–6]. As PWH age, the costs associated with managing non-HIV comorbidities and/or treatment also rise [7]. Consequently, PWH generally have higher healthcare resource utilization (HRU) rates than the general population without HIV. US-based studies have shown that PWH experience higher rates of emergency room (ER) visits, undergo more diagnostic testing per visit, have longer ER stays, and are more likely to be admitted compared to those without HIV [8].

In addition to the clinical and psychosocial impacts of HIV-1 infection on HRU, adherence to treatment is also a key factor influencing HRU. PWH who maintain very high adherence to ART (≥95%) typically experience substantially fewer all-cause or HIV-related ER visits and hospitalizations compared to those with low adherence (<80%) [9]. Despite advances, ART adherence remains challenging for some PWH [10]. Simplified single-tablet regimens, as opposed to multi-tablet regimens, are associated with higher compliance [11, 12] and lower annual HRU [9, 13]. The advent of long-acting therapies, such as bimonthly injectables, further streamlines treatment and may enhance adherence while reducing HRU [14, 15].

As HIV treatments continue to advance, identifying care gaps and understanding both the underlying disease burden and characteristics of PWH who have suboptimal ART adherence are essential. Gaining such insights may help inform the development of emerging treatment strategies that address unmet needs and ultimately enhance clinical outcomes. This retrospective study compared sociodemographic, clinical characteristics, and HRU between PWH and age- and sex-matched individuals without HIV (primary objective) in a cross-sectional analysis on 31 December 2022. However, these variables were obtained from longitudinal electronic health records using distinct lookback periods. HRU was evaluated over a 12-month lookback period (Jan–Dec 2022), while HIV diagnosis and comorbidities were assessed using a lookback period of over 2 decades. Additionally, ART treatment patterns among PWH were characterized (secondary objective) by assessing the use of ART in the 12 months preceding the assessment date (31 December 2022) or from the date of HIV diagnosis to the assessment date for those diagnosed during 2022.

## Materials and methods

Reporting of this study conforms to STrengthening the Reporting of OBservational studies in Epidemiology (STROBE) reporting guidelines [16].

### Study design and setting

The database of a large, national-level healthcare insurer (Maccabi Health Services [MHS], Israel) was utilized in this study in the context of universal healthcare coverage with access to fully subsidized ART provided by outpatient HIV centres. MHS is the second largest of 4 state-mandated healthcare funds (insurer-provider) in Israel, currently providing care to >2.7 million people (over a quarter of the population). The MHS database contains longitudinal data available since 1993, with comprehensive data since 1998, on a relatively stable population (<1% leaving MHS each year). Healthcare encounters are routinely documented in the MHS database, including comprehensive patient-level information on diagnoses, pharmacy prescriptions, and purchases as well as sociodemographic characteristics.

Longitudinal deidentified patient data from the MHS database were accessed on 6 April 2023 and extracted for analysis using a study assessment date of 31 December 2022. Overall, the data in this study span from 1998 to 2022, with specific lookback periods for different variables described in the methods. The study population focused on individuals who were alive and actively enrolled on the study assessment date.

The study was approved by the MHS institutional review board (reference number 0040–23-MHS) and was conducted in accordance with the ICH-GCP and Declaration of Helsinki, Ethical Principles for Medical Research Involving Human Subjects. Given the use of retrospective and deidentified data, a waiver of consent was granted by the institutional review board. All results consisted of aggregated data.

### Study population

Eligible PWH population met the following inclusion criteria: enrolment in MHS on the assessment date, ≥365 days of continuous enrolment prior to the assessment date, and ≥ 1 diagnostic code for HIV infection during the study period using the International Classification of Diseases, Ninth Revision, Clinical Modification (ICD-9-CM) codes (042.xx–044.xx and 795.71) and internal MHS codes. Additionally, in order to accurately identify patients with confirmed HIV infection rather than HIV exposure (eg, suspected or perinatally exposed cases), the study required 3 criteria: (1) ≥ 1 official approval for HIV treatment recorded during the study period (1998–2022; from the MHS prior medication approval centre), (2) ≥ 1 HIV treatment dispensed after approval during the last 10 years preceding the assessment date (2008–2022), and (3) an age ≥ 1 year at the last treatment date.

Controls were selected from the general population of MHS members who were enrolled on the assessment date with ≥365 days of continuous enrolment prior to the assessment date, no HIV diagnosis during the study period, and no dispensed HIV treatment/prophylaxis during the study period. For each PWH, five control individuals were randomly selected through exact matching based on age at the assessment date (5-year age groups) and sex (female/male, as recorded in the database). Socioeconomic status (SES) was not included as a matching variable, as comparing SES between PWH and controls was a study objective to better understand the relationship between SES and HIV in this setting.

### Sociodemographic and clinical characteristics

Demographic characteristics included recorded sex, age at the assessment date, and residential SES. SES was based on the score of an individual’s place of residence at the neighbourhood level, ranging from 1 (lowest) to 10 [17]. This score was originally derived by the Israel Central Bureau of Statistics using national census data and later augmented by POINTS Location Intelligence (Tel Aviv, Israel) by using aggregate data for various SES indicators (housing prices, motorization level, education, employment, and financial resources). SES was then categorized as low (1–4), medium (5, 6), or high (7–10).

The following comorbid conditions diagnosed at any time (“ever”) were identified from 1998 to 2022 using ICD-9-CM diagnosis codes and previously validated MHS chronic disease registries: any cancer, cardiovascular disease, diabetes (type 1 or type 2), chronic kidney disease, hypertension, chronic obstructive pulmonary disease, liver disease, venous thromboembolic events, and tuberculosis. History of any cancer was obtained from the National Cancer Registry and the MHS cancer registry. Cardiovascular disease was obtained from the MHS cardiovascular disease registry using diagnosis and procedural data. History of infection (‘ever’) with hepatitis C or B virus (HCV or HBV) was based on positive laboratory data from HCV RNA polymerase chain reaction and HBV surface antigen test results. Mental health comorbidities were identified using ICD-9 diagnoses and included anxiety and/or depressive disorder in the prior 12 months (ie, diagnosed and/or treated in 2022), as well as history of posttraumatic stress disorder (PTSD) and drug dependence diagnosed at any time (‘ever’). This 12-month window was used for anxiety and depression because these diagnoses were ascertained using both diagnostic codes and dispensed medications, and lifetime medication use is not sufficiently specific for these conditions. In contrast, other chronic comorbidities were assessed over an extended period to capture the patient’s historical journey. Body mass index was assessed using the most recent measure or one up to 5 years prior to the assessment date, with standard cut-offs for adults [18].

### HRU

Annual HRU was described (1 January 2022, to 31 December 2022) according to the number of patients experiencing ≥1 new hospital admission (all-cause) and the total length of stay over all hospitalizations for a given patient during the 1-year period. It also included the frequency of visits to the ER, after-hours urgent care, primary care (general practitioners/family medicine and paediatricians), specialists (all other physician specialties), and nurses.

Concomitant medication use was defined as ≥1 dispensed prescription within the 12 months prior to the assessment date. Medications were identified by the Anatomical Therapeutic Chemical code and reported in aggregate according to pharmacologic groups used in MHS.

### ART treatment patterns

Antiretroviral medications were identified using Anatomical Therapeutic Chemical codes (Supplementary Table S1). Among PWH, treatment patterns were described by the use of ≥1 dispensed ART prescription in the previous 12 months (1 January 2022 to 31 December 2022) or from the date of HIV diagnosis to the assessment date (31 December 2022) for those diagnosed in 2022. The percentage of days covered (PDC) by ART was defined as the number of days with a dispensed ART prescription divided by the total number of days in the observation period (365 days, or for PWH diagnosed with HIV in 2022, the number of days between diagnosis and 31 December 2022) [19]. PDC was measured as a continuous variable and reported as PDC ≥90% vs. <90% [20]. Treatment gaps in ART use were examined, with ‘stable use’ denoting ≤5-day gap between the end-of-supply days of 1 ART prescription fill and the date of the following fill. ‘Interrupted use’ denoted a > 5-day and < 90-day gap between end-of-supply days of 1 fill and the date of the following fill. ‘Discontinued’ denoted ≥90 days from the end-of-supply days of the last fill until the study assessment date.

### Statistical analyses

Descriptive statistics included numbers and percentages for dichotomous and categorical variables. Depending on their distribution, continuous variables were summarized as mean and SD or median and range and/or interquartile range (IQR). Demographic and clinical characteristics and HRU were compared between PWH and matched controls, with results stratified by recorded sex. PWH were also compared based on their ART treatment patterns (PDC ≥90% vs. <90% and discontinuation vs. no discontinuation). Missing data were included in analyses as a category. Comparisons were made using the Chi-square test, Student *t*-test, or Wilcoxon rank-sum test, depending on the data characteristics. Standardized mean differences were reported. Statistical significance was set at *P* < 0.05, and analyses were performed using R (version 4.0.2, R Foundation for Statistical Computing, Vienna).

## Results

**Figure 1.**
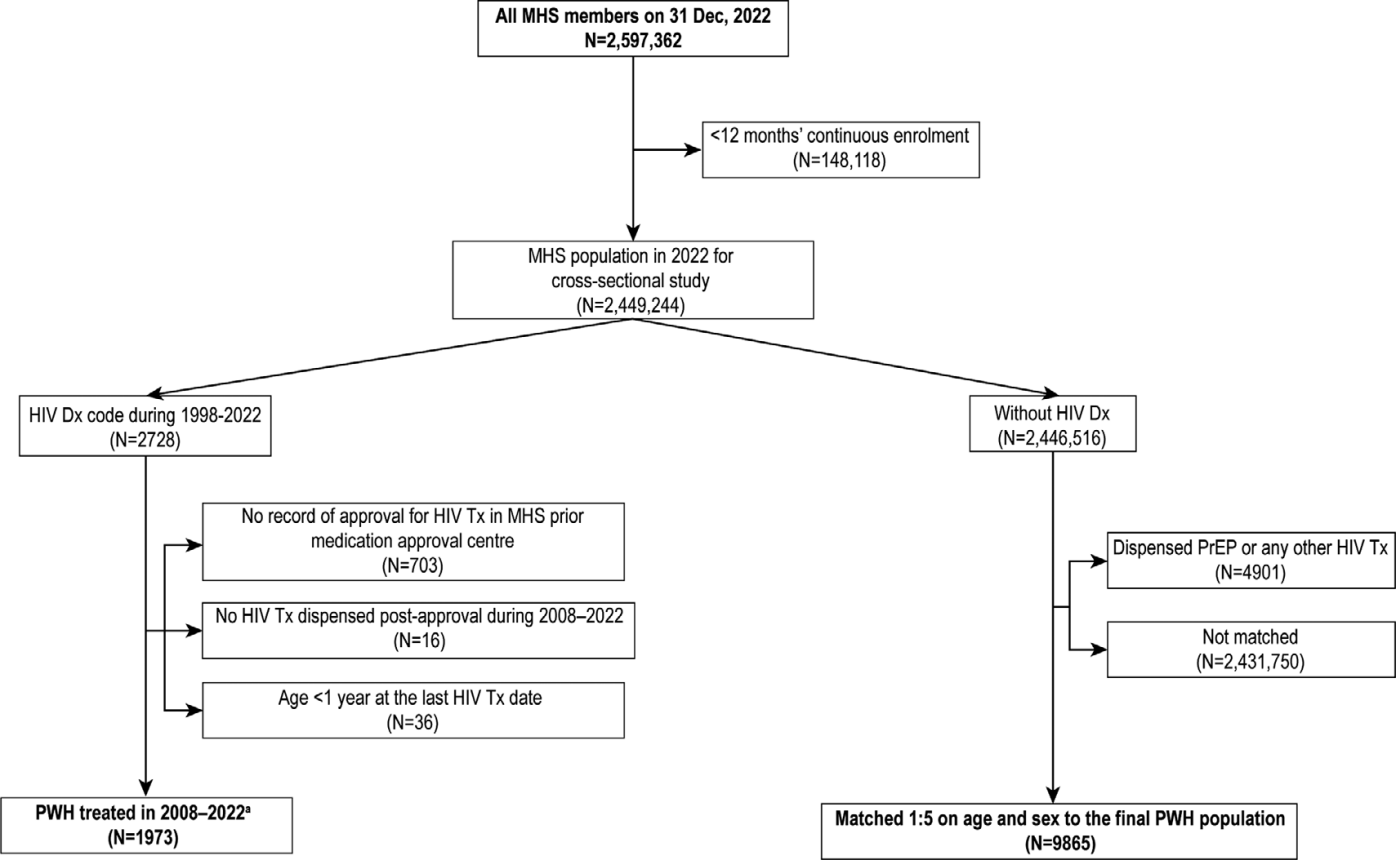
Selection of the study population: PWH and age- and sex-matched controls without HIV (31 December 2022; MHS). *Note*: ^a^Main study population of PWH. Dx, diagnosis; MHS, Maccabi Health Services; PWH, people with HIV; PrEP, preexposure prophylaxis; Tx, treatment.

Of the 2.6 million members of MHS recorded on the assessment date (Figure 1), 1973 PWH were included in this study. The control group included 9,865 individuals without an HIV diagnosis who were age- and sex-matched with the PWH group. The mean age (48 years) and age-group distribution were similar between PWH and controls, and both groups had 74% males.

### PWH and matched general population without HIV

#### Sociodemographic and clinical characteristics

PWH were less likely to reside in high (7–10) or low (1–3) SES areas than controls (Table 1). PWH had a significantly higher prevalence of mental health issues, including anxiety and/or depression (26.2% vs. 13.5%, P < 0.001), PTSD (3.4% vs. 2.5%, P < 0.026), and drug dependence (8.0% vs. 0.5%, P < 0.001) compared with controls. The prevalence of several other comorbid conditions was significantly higher among PWH vs. matched controls (P < 0.001), including liver disease (20.9% vs. 12.5%), chronic kidney disease (14.9% vs. 7.3%), any cancer (7.2% vs. 5.3%), HCV infection (5.6% vs. 0.1%), venous thromboembolic events (4.7% vs. 2.5%), tuberculosis (3.6% vs. 0.4%), and HBV infection (3.4% vs. 0.4%). The prevalence of hypertension, cardiovascular disease, and chronic obstructive pulmonary disease was similar between the two groups.

**Table 1.** Sociodemographic and clinical characteristics of PWH and age- and sex-matched controls without HIV

	PWH(*N* = 1973)^a^	Matched controls(*N* = 9,865)^a^	Difference^b^	*P* value^c^
***Sociodemographic and clinical characteristics on 31 December 2022***				
**Mean age ± SD, y**	48.2 ± 11.2	48.7 ± 11.2	−0.05	0.093
Median (IQR)	48.1 (40.5, 55.2)	49.0 (41.3, 55.2)		
**Age group, y**			0	>0.9
<18	7 (0.4)	34 (0.3)		
18–34	215 (10.9)	1,076 (10.9)		
35–49	904 (45.8)	4,520 (45.8)		
50–74	827 (41.9)	4,135 (41.9)		
75+	20 (1.0)	100 (1.0)		
**Males**	1,467 (74.4)	7,335 (74.4)	0	>0.9
**Residential SES**			0.29	<0.001
Low (1–3)	219 (11.1)	1,616 (16.4)		
Mid (4–6)	968 (49.1)	3,468 (35.2)		
High (7–10)	782 (39.6)	4,731 (48.0)		
Missing	4 (0.2)	50 (0.5)		
**BMI**			0.51	<0.001
Normal	842 (42.7)	2,314 (23.5)		
Underweight	68 (3.4)	106 (1.1)		
Overweight	532 (27.0)	2,883 (29.2)		
Obese	242 (12.3)	2053 (20.8)		
Missing	289 (14.6)	2,509 (25.4)		
***Comorbidities* ^d^**				
**Cancer**	142 (7.2)	523 (5.3)	0.08	<0.001
**Cardiovascular disease^e^**	145 (7.3)	802 (8.1)	−0.03	0.2
**Diabetes**	139 (7.0)	924 (9.4)	−0.08	<0.001
**Chronic kidney disease**	293 (14.9)	723 (7.3)	0.24	<0.001
**Hypertension**	329 (16.7)	1,577 (16.0)	0.02	0.4
**COPD**	37 (1.9)	138 (1.4)	0.04	0.11
**Liver disease^f^**	412 (20.9)	1,235 (12.5)	0.23	<0.001
**HCV PCR positive**	111 (5.6)	10 (0.1)	0.34	<0.001
**HBV sAg positive**	67 (3.4)	36 (0.4)	0.22	<0.001
**VTE**	93 (4.7)	248 (2.5)	0.12	<0.001
**Tuberculosis**	71 (3.6)	35 (0.4)	0.23	<0.001
**Depression and/or anxiety**	516 (26.2)	1,333 (13.5)	0.32	<0.001
**PTSD**	67 (3.4)	248 (2.5)	0.05	0.026
**Drug dependence**	157 (8.0)	45 (0.5)	0.38	<0.001

a *n* (%), unless stated otherwise.

b Standardized mean difference.

c Wilcoxon rank sum test, Pearson chi-squared test, and Fisher exact test.

d Ever diagnosed for all comorbidities except for depression and/or anxiety, which was diagnosed and/or treated in the past 12 months (2022).

e Included ischemic heart disease/myocardial infarction, congestive heart failure, peripheral vascular disease, cerebrovascular disease, atrial fibrillation, cerebrovascular atherosclerosis, valve disease, cardiomyopathies, and rhythm disorders.

f Mild, moderate, or severe.

BMI, body mass index; COPD, chronic obstructive pulmonary disease; HBVsAg, hepatitis B virus surface antigen; HCV PCR, hepatitis C virus polymerase chain reaction; IQR, interquartile range; PTSD, posttraumatic stress disorder; PWH, people with HIV; SES, socioeconomic status; VTE, venous thromboembolic event.

#### HRU

PWH showed consistently higher annual HRU (Jan–Dec 2022) than controls (Table 2). The rate of ≥1 new hospital admissions per year among PWH was 11.4% vs. 6.6% among controls (P < 0.001). The total annual length of stay was also longer among PWH vs. controls, with a median (IQR) of 4 (2, 12) vs. 3 (1, 6) days (mean ± SD: 14.9 ± 32.3 vs. 7.9 ± 21.4, respectively; P < 0.001). PWH were more likely to visit all types of healthcare services at least once per year (Table 2) and had a higher annual frequency of visits to all services, except specialist and after-hours urgent care (Supplementary Table S2). Annual concomitant medications use in 2022 was higher among PWH than controls (P < 0.05) for antibiotic (46.5% vs. 33.7%), gastrointestinal (28.8% vs. 19.7%), antidepressant (21.3% vs. 11.1%), cardiovascular disease (18.8% vs. 16.0%), antipsychotic (6.3% vs. 2.7%), and diuretic (2.6% vs. 1.6%) agents but not for hypolipidemic, antithrombotic, antidiabetic, or respiratory system medications. When results were stratified by sex, females with HIV had a higher prevalence of hypertension, liver disease, and psychiatric disorders with a similar prevalence of cancer, diabetes, venous thromboembolic events, and PTSD than controls (Supplementary Table S3). Among males with HIV, the prevalence of cancer, liver disease, and psychiatric disorders was higher while that of hypertension and PTSD was similar compared with controls.

**Table 2.** Annual HRU (2022) among PWH vs age- and sex-matched controls without HIV

HRU in 2022	PWH (*N* = 1973)^a^	Matched controls (*N* = 9,865)^a^	Difference^b^	*P* value^c^
Hospitalization, ≥1 new admission	224 (11.4)	650 (6.6)	0.17	<0.001
Emergency department, ≥1 visit	522 (26.5)	1879 (19.0)	0.18	<0.001
After-hours urgent care, ≥1 visit	83 (4.2)	305 (3.1)	0.06	0.011
Primary care, ≥1 visit	1923 (97.5)	8,953 (90.8)	0.29	<0.001
Specialists, ≥1 visit	1,392 (70.6)	6,734 (68.3)	0.05	0.045
Nursing, ≥1 visit	1,393 (70.6)	4,781 (48.5)	0.46	<0.001

a *n* (%).

b Standardized mean difference.

c Wilcoxon rank sum test, Pearson chi-squared test, and Fisher exact test.

HRU, healthcare resource utilization; PWH, people with HIV.

### HIV treatment patterns among PWH

**Figure 2.**
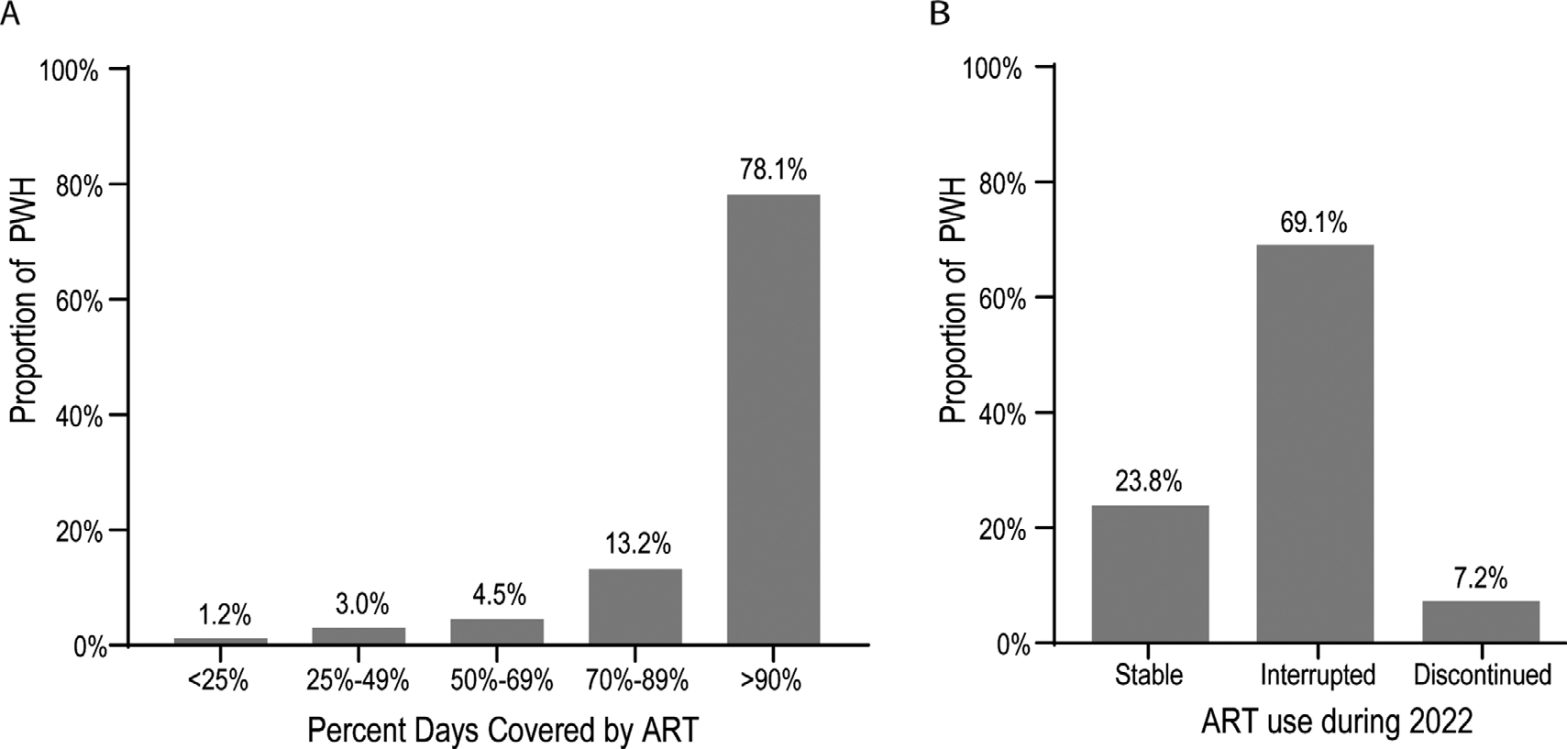
ART treatment patterns based on (a) PDC and (b) discontinuation and interruption among PWH with ≥ 1 ART dispense date in 2022 (n = 1907). *Note*: Stable use, ≤5-day gap between prescriptions; interrupted use, >5-day and < 90-day gap between prescriptions; and discontinued, ≥90 days from last prescription to 31 December 2022. ART, antiretroviral therapy; PDC, percentage of days covered; PWH, people with HIV.

Among the full PWH study population (n = 1973), the median (IQR) time on ART was 8.5 (4.7, 12.2, range 0–16) years. The most recent ART dispensing occurred at a median (IQR) of 39.0 (19–67) days (range, 1–3,464) before the assessment date. The majority of PWH (n = 1907, 96.7%) had ART dispensed at least once in 2022, with a median (IQR) of 4 (4–6, range 0–17) ART dispensations, each typically containing a 1-month ART supply. PDC of ≥90% was observed among 78.1% of PWH (Figure 2a). However, 69.1% of PWH had an interruption in ART dispensations (>5-day and < 90-day gap between end-of-supply days to the next dispense date), and 7.2% discontinued ART (≥90 days since the end-of-supply days of the last ART prescription up to 31 December 2022) (Figure 2b).

### Clinical characteristics and HRU among PWH based on treatment patterns

In 2022, there were 1770 (92.8%) PWH without ART discontinuation (including stable use [≤5-day gap] and interruption [5-day and < 90-day gap]) and 137 (7.2%) PWH with ART discontinuation (≥90 days from last prescription to 31 December 2022). PWH with discontinuation were younger than those without discontinuation, with the majority aged 35–49 years (53.3% vs. 45.1% for those without discontinuation) (Table 3). Discontinuation was associated with a higher prevalence of drug dependence and a lower percentage of individuals with ≥1 specialist or nurse visit per year. Depression and/or anxiety diagnosed and/or treated in the past 12 months was not associated with ART discontinuation (Table 3), while ever diagnosed (1998–2022) depression and/or anxiety was more prevalent among PWH who discontinued ART in 2022 vs. those who did not (65.0% vs. 54.9%; *P* < 0.05).

**Table 3.** Sociodemographic and clinical characteristics and HRU of PWH by ART discontinuation in the past 12 months (2022)

	Without ART discontinuation^a^ (*n* = 1770)^b^	With ART discontinuation^a^ (*n* = 137)^b^	Difference^c^	*P* value^d^
***Sociodemographic and clinical characteristics on 31 December 2022***				
**Mean age (SD), y**	48.5 (11.2)	44.7 (10.7)	0.35	<0.001
Median age (IQR)	48.4 (40.7, 55.7)	45.5 (38.9, 52.1)		
**Age group, y**			0.29	0.004
<18	4 (0.2)	3 (2.2)		
18–34	189 (10.7)	17 (12.4)		
35–49	799 (45.1)	73 (53.3)		
50–74	759 (42.9)	43 (31.4)		
75+	19 (1.1)	1 (0.7)		
**Males**	1,314 (74.2)	105 (76.6)	0.06	0.5
**Residential SES**			0.16	0.5
Low (1–3)	202 (11.4)	12 (8.8)		
Mid (4–6)	857 (48.4)	76 (55.5)		
High (7–10)	707 (39.9)	49 (35.8)		
Missing	4 (0.2)	0 (0.0)		
**BMI**			0.18	0.4
Normal	757 (42.8)	63 (46.0)		
Underweight	60 (3.4)	6 (4.4)		
Overweight	489 (27.6)	35 (25.5)		
Obese	224 (12.7)	11 (8.0)		
Missing	240 (13.6)	22 (16.1)		
***Comorbidities*** ^e^				
**Cancer**	132 (7.5)	7 (5.1)	0.1	0.3
**Cardiovascular disease**	137 (7.7)	6 (4.4)	0.14	0.2
**Diabetes**	131 (7.4)	6 (4.4)	0.13	0.2
**Chronic kidney disease**	277 (15.6)	11 (8.0)	0.24	0.016
**Hypertension**	309 (17.5)	12 (8.8)	0.26	0.009
**COPD**	36 (2.0)	1 (0.7)	0.11	0.5
**Liver disease^f^**	368 (20.8)	35 (25.5)	−0.11	0.2
**HCV PCR positive**	95 (5.4)	12 (8.8)	−0.13	0.1
**HBVsAg positive**	58 (3.3)	7 (5.1)	−0.09	0.2
**VTE**	84 (4.7)	6 (4.4)	0.02	0.8
**Tuberculosis**	60 (3.4)	8 (5.8)	−0.12	0.15
**Depression and/or anxiety**	465 (26.3)	43 (31.4)	−0.11	0.2
**PTSD**	60 (3.4)	7 (5.1)	−0.09	0.3
**Drug dependence**	130 (7.3)	20 (14.6)	−0.23	0.002
***HRU in 2022***				
**Hospitalization, ≥1 new admission**	202 (11.4)	15 (10.9)	0.01	0.9
**Emergency department, ≥1 visit**	472 (26.7)	36 (26.3)	0.01	>0.9
**After-hours urgent care, ≥1 visit**	76 (4.3)	5 (3.6)	0.03	0.7
**Primary care, ≥1 visit**	1752 (99.0)	133 (97.1)	0.14	0.068
**Specialists, ≥1 visit**	1,293 (73.1)	82 (59.9)	0.28	<0.001
**Nursing, ≥1 visit**	1,287 (72.7)	87 (63.5)	0.2	0.021

a Defined as a > 90-day gap between the end of the last ART prescription and the assessment date.

b *n* (%), unless stated otherwise.

c Standardized mean difference.

d Wilcoxon rank sum test, Pearson chi-squared test, and Fisher exact test.

e Ever diagnosed for all comorbidities except for depression and/or anxiety, which was diagnosed and/or treated in the past 12 months (2022).

f Mild, moderate, or severe.

ART, antiretroviral therapy; BMI, body mass index; COPD, chronic obstructive pulmonary disease; HBVsAg, hepatitis B virus surface antigen; HCV PCR, hepatitis C virus polymerase chain reaction; HRU, healthcare resources utilization; IQR, interquartile range; PTSD, posttraumatic stress disorder; PWH, people with HIV; SES, socioeconomic status; VTE, venous thromboembolic event.

## Discussion

This retrospective study shows a higher prevalence of comorbidities and HRU among PWH compared to age- and sex-matched controls without HIV within the Israeli healthcare system, where ART is subsidized, and the most current regimens are available. The prevalence of anxiety and/or depression, PTSD, drug dependence, liver disease, chronic kidney disease, and cancer was higher among PWH. PWH were also more likely to be hospitalized or to visit ER, after-hours urgent care, primary care, specialists, or nurses at least once per year. These findings in an Israeli population (representative of the entire national population with a tendency towards residents of the central districts) demonstrate that PWH experience a disproportionate burden of comorbidities and elevated HRU compared to the general population and align with other recent real-world evidence studies [6, 8, 21–23]. While modern ART has greatly improved disease management and long-term outcomes, persistent challenges remain, such as higher rates of mental health conditions, chronic organ diseases, and increased hospitalizations. This study underscores ongoing gaps in care and signals a need for targeted interventions that address both medical and psychosocial aspects, ensuring that evolving treatment strategies better meet the complex needs of PWH.

In our study, SES was determined by the geographic area linked to the individual’s home address rather than their SES. The finding that most controls resided in regions of lower or higher SES, while most PWH were from mid-range areas, contrasts with typical patterns where lower SES often correlates with increased HIV risk. In Israel, this distribution likely reflects distinct sociocultural and religious influences, as highly religious communities that occupy the lowest SES regions are largely protected from HIV through behavioural norms [24]. Additionally, individuals with higher education and resources tend to choose high-performing health coverage funds and undergo earlier testing, whereas groups at greatest social disadvantage, such as migrants and people who use drugs, may be underrepresented within the MHS insurance coverage. These factors suggest that HIV risk in Israel is shaped by a complex interplay of religious, behavioural, and health system dynamics, rather than SES alone.

Unlike prior findings of increased cardiovascular disease and hypertension among PWH [25, 26], our findings indicate comparable prevalence of these conditions between PWH and controls. This notable divergence offers a novel perspective on comorbidities among PWH. Although not specifically examined in our study, earlier HIV diagnosis, prompt initiation of ART, and the use of safer treatment regimens in our cohort may have played a role in reducing cardiovascular risk. Additionally, the lower likelihood of obesity observed in PWH in our sample could contribute to reduced rates of obesity-related comorbidities and affect overall cardiovascular risk profiles. Another possibility could be an increased healthcare engagement, including more frequent healthcare interactions between Israeli PWH and providers for routine monitoring and treatment reviews, providing opportunities for better management of ART and/or HIV-related comorbidities, which is likely influenced by cultural, healthcare system structure, and public health policies unique to Israel. Notably, we did observe an increased burden of hypertension among women with HIV vs. control women, highlighting an important sex-specific disparity. Taken together, these findings highlight the importance of considering population-specific factors, healthcare engagement, and sex when interpreting comorbidity outcomes among PWH.

While the current study did not evaluate the cost of healthcare, other studies have found that mean annual overall healthcare costs are at least 2-fold and up to 6- to 7-fold higher for PWH than people without HIV [6, 21–23]. The cost of ART is generally the largest component of healthcare costs [21, 22], but excess costs among PWH are also associated with non-ART medications, specialist and primary care visits, laboratory tests, emergency department visits, hospitalizations, and intensive care visits [21–23]. Therefore, continued characterization of the burden of comorbidities and associated healthcare resource utilization among PWH as they age will be of utmost importance.

In the current study, PWH had higher annual use of healthcare resources across several services, with nearly double the rate of at least 1 new hospital admission. A similar rate of hospitalizations and a 2-fold higher mean annual per-patient hospitalization costs were observed among PWH vs. controls in a French claims database study [21]. In addition to hospitalizations, the use of any healthcare service at least once per year was significantly higher among PWH than in the control group in this study. Notably, the frequency of visits per year was higher in primary care among PWH, but not in specialist urgent care. Similar findings were reported in France, with a higher mean number of primary care visits among PWH vs. controls [21]. It is important to note that in Israel, dedicated HIV centres, catering specifically to PWH, are spread throughout the country. They offer secondary and primary care services to this population as part of subsidized healthcare coverage. Often, following a recommendation from a specialized physician at an HIV centre, PWH will follow up with their primary care physician for ongoing care. This may, in part, account for the higher frequency of PWH seen in primary care.

An estimated PDC of <90% was observed in approximately 1 in 5 PWH (~22%) in the current study, a proportion lower than that reported in US studies, which ranged from 34.6% to 61.3% [9, 10, 27–29]. Although the rate of treatment discontinuation observed here (7.2%) was higher than expected for this setting with state-funded access to ART, it was still similar to estimates of annual ART discontinuation in two US studies that ranged from 5.6% to 10.1% [27, 30]. Discontinuation was associated with younger age, higher prevalence of some mental health challenges, and lower utilization of specialist and nurse services. A high proportion (45%) of treatment interruption (7- to <90-day gap) has been documented in the United States [27] as well as in this study (69%), suggesting that short-term treatment interruptions may be more prevalent than potentially presumed based on clinical observations of ongoing successful viral suppression among PWH. Studies have found an association between mental health challenges and suboptimal adherence [27, 28, 31], highlighting an opportunity to enhance adherence support. Some strategies for improving and maintaining adherence include identifying current barriers to ongoing adherence and providing a constructive, nonjudgmental, and collaborative approach to problem-solving [32]. A trusting relationship between the individual with HIV and their principal healthcare provider is essential. Simplifying ART regimens or reducing side effects, while still maintaining a high barrier to viral resistance, can be complemented by connecting PWH to additional supports that address unmet socioeconomic or mental health needs.

Key strengths of this study include its large sample of PWH and the use of age- and sex-matched controls without HIV, all drawn from the same health maintenance organization (ie, MHS) that provides both insurance and healthcare services. This approach enables a robust and comprehensive analysis of sociodemographic, clinical, and treatment characteristics across a wide range of data points rarely examined together in a single study. Nearly 8,400 people (2021 data) are registered as living with HIV in Israel [33], and this study, using MHS data, captured a significant and representative portion of that population. The study dataset is relatively current and potentially also applicable to other systems with similar access and care provision in this therapeutic context. The limitations of this study include the relatively higher income level of the MHS population and the lack of assessment of participants’ place of birth or gender identity. Laboratory data (HIV-1 RNA levels and CD4 cell counts) were unavailable for this analysis, and identification of PWH was based solely on diagnostic codes. The mental health burden may be underestimated in this study population due to diagnostic and stigma-related challenges and because mental health clinic notes are often recorded as free text in patient records, which were not analysed in this study. Future studies may examine free-text data using recent large language models, allowing for an in-depth analysis of physician notes. Analysis of ART adherence depended on supply-day data entered by the physician, and different coding practices for combination treatments may have over- or underestimated supply days (which could affect estimates of PDC and treatment gaps), or PWH may have accessed ART through other means. The PDC cut-off may have also led to an underestimation of adherence. Discontinuation may also be underestimated among PWH newly diagnosed in 2022 (n = 73). Additionally, in some healthcare systems (including MHS-related), prescription renewals may be coded as primary care visits, and thus, these visits warrant further investigation. Finally, causes of suboptimal ART adherence and interruptions, such as difficulty swallowing pills, concern over inadvertent disclosure of HIV status, or pill fatigue, were not examined as these data were not available. Further research, including qualitative studies, is warranted to better understand the reasons and possible support for suboptimal adherence in this population.

## Conclusions

In summary, even with the availability of state-funded, modern ART regimens, PWH continue to bear a greater burden of comorbidities and elevated HRU compared to those without HIV. This underscores persistent gaps that go beyond access to medication, including the need for improved support systems to promote treatment adherence and address the psychosocial challenges faced by PWH. The frequent interruptions and discontinuations in therapy highlight how existing healthcare models may not fully meet the complex needs of this population. Future advances in HIV care should focus on holistic strategies, such as therapies that reduce chronic inflammation and comorbidities, simplify adherence requirements, and provide durable viral suppression to improve long-term health, quality of life, and progress toward a cure. Continued research into novel therapeutic approaches and integrated care models remains essential to address both the biomedical and social dimensions of living with HIV.

## Supporting information

10.1017/S0950268826101265.sm001 Weil et al. supplementary material Weil et al. supplementary material

## Data Availability

Data are available from the authors upon request.

## Supplementary material

The supplementary material for this article can be found at http://doi.org/10.1017/S0950268826101265.
