# Supplementary material for: Burden of comorbid disease, treatment, and healthcare utilization among people with HIV: A retrospective, cross-sectional study from a large healthcare organization
Source: Epidemiol Infect. 2026 Mar 24;154:e45. doi: 10.1017/S0950268826101265 (PMC13100925; doi:10.1017/S0950268826101265)
Supplement: Weil et al. supplementary material [file S0950268826101265sup001.docx]

**Supplementary Material**

**Table S1. Anatomical Therapeutic Chemical (ATC) codes for antiretrovirals.^a,b^**

| **Class** | **ATC** | **ATC Description** | **Acronyms** |
| --- | --- | --- | --- |
| PI | J05AE03 | ritonavir | RTV |
| PI | J05AE08 | atazanavir | ATV |
| PI | J05AE10 | darunavir | DRV |
| NRTI | J05AF01 | zidovudine | ZDV or AZT |
| NRTI | J05AF05 | lamivudine | 3TC |
| NRTI | J05AF06 | abacavir | ABC |
| NRTI | J05AF07 | tenofovir disoproxil | TDF |
| NNRTI | J05AG01 | nevirapine | NVP |
| NNRTI | J05AG03 | efavirenz | EFV |
| NNRTI | J05AG04 | etravirine | ETR |
| NNRTI | J05AG05 | rilpivirine | RPV |
| NNRTI | J05AG06 | doravirine | DOR |
| INSTI | J05AJ01 | raltegravir | RAL |
| INSTI | J05AJ03 | dolutegravir | DTG |
| INSTI | J05AJ04 | cabotegravir | CAB |
| Comb | J05AR01 | lamivudine/zidovudine | 3TC/ZDV |
| Comb | J05AR02 | abacavir sulfate/lamivudine | ABC/3TC |
| Comb | J05AR03 | emtricitabine/tenofovir DF | FTC/TDF |
| Comb | J05AR06 | efavirenz/emtricitabine/tenofovir DF | EFV/FTC/TDF |
| Comb | J05AR08 | emtricitabine/rilpivirine hydrochloride/tenofovir disoproxil fumarate | FTC/RPV/TDF |
| Comb | J05AR09 | elvitegravir, cobicistat, emtricitabine, and tenofovir disoproxil fumarate | EVG/COBI/FTC/TDF |
| Comb | J05AR10 | lopinavir/ritonavir | LPV/RTV |
| Comb | J05AR13 | abacavir sulfate/dolutegravir sodium/lamivudine | ABC/DTG/3TC |
| Comb | J05AR14 | darunavir/cobicistat | DRV/COBI |
| Comb | J05AR18 | elvitegravir/cobicistat/emtricitabine/tenofovir alafenamide fumarate | EVG/COBI/FTC/TAF |
| Comb | J05AR20 | bictegravir/emtricitabine/tenofovir alafenamide | BIC/FTC/TAF |
| Comb | J05AR21 | dolutegravir sodium/rilpivirine hydrochloride | DTG/RPV |
| Comb | J05AR24 | dolutegravir sodium/lamivudine | DTG/3TC |
| Comb | J05AR25 | doravirine/lamivudine/tenofovir DF | DOR/3TC/TDF |
| CCR5 antagonists | J05AX09 | maraviroc | MVC |
| Capsid inhibitors | J05AX31 | lenacapavir | LEN |
| ^a^Adapted to this study based on https://hivinfo.nih.gov/understanding-hiv/fact-sheets/fda-approved-hiv-medicines. Listed Anatomical Therapeutic Chemical (ATC) codes include antiretrovirals captured in the main analysis (2018−2022). This list was expanded to include older treatments to determine the earliest date of dispensed ART.  ^b^HIV Pre-Exposure Prophylaxis Medications examined included nucleoside reverse transcriptase inhibitor (Truvada; emtricitabine/tenofovir DF [FTC/TDF]).  Comb, combination therapy; INSTI, integrase strand transfer inhibitor; NNRTI, non-nucleoside reverse transcriptase inhibitor; NRTI, nucleoside reverse transcriptase inhibitor; PI, protease inhibitor. | | | |

**Table S2. Number of healthcare visits (2022) among PWH vs age- and sex-matched controls without HIV.**

| **HRU in 2022** | **PWH**  (N=1973) | **Matched controls** (N=9865) | **Difference**^a^ | | ***P* value**^b^ |
| --- | --- | --- | --- | --- | --- |
| **Emergency department^c^** |  |  | | 0.17 | 0.001 |
| n | 522 | 1879 | |  |  |
| Median (IQR) | 1.0 (1.0, 2.0) | 1.0 (1.0, 2.0) | |  |  |
| Range | 1, 13 | 1, 13 | |  |  |
| **After-hours urgent care^c^** |  |  | | 0.22 | 0.1 |
| n | 83 | 305 | |  |  |
| Median (IQR) | 1.0 (1.0, 2.0) | 1.0 (1.0, 1.0) | |  |  |
| Range | 1, 4 | 1, 5 | |  |  |
| **Primary care^c^** |  |  | | 0.45 | <0.001 |
| n | 1923 | 8953 | |  |  |
| Median (IQR) | 10.0 (7.0, 16.0) | 7.0 (4.0, 12.0) | |  |  |
| Range | 1, 64 | 1, 85 | |  |  |
| **Specialists^c^** |  |  | | 0.01 | 0.9 |
| n | 1392 | 6734 | |  |  |
| Median (IQR) | 3.0 (2.0, 6.0) | 3.0 (2.0, 6.0) | |  |  |
| Range | 1, 53 | 1, 61 | |  |  |
| **Nursing^c^** |  |  | | 0.11 | <0.001 |
| n | 1393 | 4781 | |  |  |
| Median (IQR) | 2.0 (1.0, 4.0) | 2.0 (1.0, 3.0) | |  |  |
| Range | 1, 177 | 1, 60 | |  |  |
| ^a^Standardized mean difference. | | | | | |
| ^b^Wilcoxon rank sum test, Pearson’s chi-squared test, and Fisher’s exact test.  ^c^Medians and ranges were reported only among those who used a given healthcare resource; therefore, all ranges start at 1 (exclude zeros).  HRU, healthcare resource utilization; IQR, interquartile range; PWH, people with HIV. | | | | | |

**Table S3. Sociodemographic and clinical characteristics and HRU of PWH stratified by sex.**

|  | **Females** | | | | **Males** | | | |
| --- | --- | --- | --- | --- | --- | --- | --- | --- |
|  | **PWH**  (N=506)^a^ | **Matched controls**  (N=2530)^a^ | **Difference**^b^ | ***P* value**^c^ | **PWH**  (N=1467)^a^ | **Matched controls** (N=7335)^a^ | **Difference**^b^ | ***P* value**^c^ |
| ***Sociodemographic and clinical characteristics on December 31, 2022*** | | | | | | | | |
| **Mean age ± SD, y** | 47.6±10.5 | 48.0±10.7 | -0.04 | 0.4 | 48.4±11.4 | 48.9±11.4 | −0.05 | 0.14 |
| Median (IQR) | 47.5 (40.8, 53.6) | 48.7 (41.9, 54.3) |  |  | 48.4 (40.2, 55.8) | 49.0 (41.0, 55.8) |  |  |
| **Age group, y** |  |  | 0.01 | >0.9 |  |  | 0.01 | >0.9 |
| <18 | 5 (1.0) | 22 (0.9) |  |  | 2 (0.1) | 12 (0.2) |  |  |
| 18–34 | 33 (6.5) | 168 (6.6) |  |  | 182 (12.4) | 908 (12.4) |  |  |
| 35–49 | 271 (53.6) | 1355 (53.6) |  |  | 633 (43.1) | 3165 (43.1) |  |  |
| 50–74 | 195 (38.5) | 975 (38.5) |  |  | 632 (43.1) | 3160 (43.1) |  |  |
| 75+ | 2 (0.4) | 10 (0.4) |  |  | 18 (1.2) | 90 (1.2) |  |  |
| **Residential SES** |  |  | 0.58 | <0.001 |  |  | 0.23 | <0.001 |
| Low (1–3) | 82 (16.2) | 511 (20.2) |  |  | 137 (9.3) | 1105 (15.1) |  |  |
| Mid (4–6) | 309 (61.1) | 877 (34.7) |  |  | 659 (44.9) | 2591 (35.3) |  |  |
| High (7–10) | 115 (22.7) | 1130 (44.7) |  |  | 667 (45.5) | 3601 (49.1) |  |  |
| Missing | 0 (0.0) | 12 (0.5) |  |  | 4 (0.3) | 38 (0.5) |  |  |
| **BMI** |  |  | 0.33 | <0.001 |  |  | 0.58 | <0.001 |
| Normal | 196 (38.7) | 737 (29.1) |  |  | 646 (44.0) | 1577 (21.5) |  |  |
| Underweight | 30 (5.9) | 52 (2.1) |  |  | 38 (2.6) | 54 (0.7) |  |  |
| Overweight | 119 (23.5) | 634 (25.1) |  |  | 413 (28.2) | 2249 (30.7) |  |  |
| Obese | 91 (18.0) | 598 (23.6) |  |  | 151 (10.3) | 1455 (19.8) |  |  |
| Missing | 70 (13.8) | 509 (20.1) |  |  | 219 (14.9) | 2000 (27.3) |  |  |
| ***Comorbidities***^d^ |  |  |  |  |  |  |  |  |
| **Cancer** | 21 (4.2) | 156 (6.2) | −0.09 | 0.077 | 121 (8.2) | 367 (5.0) | 0.13 | <0.001 |
| **Cardiovascular disease** | 22 (4.3) | 81 (3.2) | 0.06 | 0.2 | 123 (8.4) | 721 (9.8) | −0.05 | 0.086 |
| **Diabetes** | 35 (6.9) | 156 (6.2) | 0.03 | 0.5 | 104 (7.1) | 768 (10.5) | −0.12 | <0.001 |
| **Chronic kidney disease** | 78 (15.4) | 158 (6.2) | 0.3 | <0.001 | 215 (14.7) | 565 (7.7) | 0.22 | <0.001 |
| **Hypertension** | 96 (19.0) | 336 (13.3) | 0.16 | <0.001 | 233 (15.9) | 1241 (16.9) | −0.03 | 0.3 |
| **COPD** | 7 (1.4) | 20 (0.8) | 0.06 | 0.2 | 30 (2.0) | 118 (1.6) | 0.03 | 0.2 |
| **Liver disease^e^** | 104 (20.6) | 262 (10.4) | 0.28 | <0.001 | 308 (21.0) | 973 (13.3) | 0.21 | <0.001 |
| **HCV PCR positive** | 32 (6.3) | 2 (0.1) | 0.36 | <0.001 | 79 (5.4) | 8 (0.1) | 0.33 | <0.001 |
| **HBVsAg positive** | 13 (2.6) | 9 (0.4) | 0.19 | <0.001 | 54 (3.7) | 27 (0.4) | 0.24 | <0.001 |
| **VTE** | 17 (3.4) | 94 (3.7) | −0.02 | 0.7 | 76 (5.2) | 154 (2.1) | 0.17 | <0.001 |
| **Tuberculosis** | 23 (4.5) | 8 (0.3) | 0.28 | <0.001 | 48 (3.3) | 27 (0.4) | 0.22 | <0.001 |
| **Depression and/or anxiety** | 124 (24.5) | 447 (17.7) | 0.17 | <0.001 | 392 (26.7) | 886 (12.1) | 0.38 | <0.001 |
| **PTSD** | 13 (2.6) | 54 (2.1) | 0.03 | 0.5 | 54 (3.7) | 194 (2.6) | 0.06 | 0.029 |
| **Drug dependence** | 26 (5.1) | 2 (0.1) | 0.32 | <0.001 | 131 (8.9) | 43 (0.6) | 0.4 | <0.001 |
| ***HRU in 2022*** |  |  |  |  |  |  |  |  |
| **Hospitalization, ≥1 new admission** | 64 (12.6) | 165 (6.5) | 0.21 | <0.001 | 160 (10.9) | 485 (6.6) | 0.15 | <0.001 |
| **Emergency department, ≥1 visit** | 122 (24.1) | 522 (20.6) | 0.08 | 0.081 | 400 (27.3) | 1357 (18.5) | 0.21 | <0.001 |
| **After-hours urgent care, ≥1 visit** | 27 (5.3) | 60 (2.4) | 0.15 | <0.001 | 56 (3.8) | 245 (3.3) | 0.03 | 0.4 |
| **Primary care, ≥1 visit** | 496 (98.0) | 2407 (95.1) | 0.16 | 0.004 | 1427 (97.3) | 6546 (89.2) | 0.32 | <0.001 |
| **Specialists, ≥1 visit** | 410 (81.0) | 2061 (81.5) | –0.01 | 0.8 | 982 (66.9) | 4673 (63.7) | 0.07 | 0.018 |
| **Nursing, ≥1 visit** | 327 (64.6) | 1336 (52.8) | 0.24 | <0.001 | 1066 (72.7) | 3445 (47.0) | 0.54 | <0.001 |
| ^a^n (%), unless otherwise stated. | | | | | | | | |
| ^b^Standardized mean difference. | | | | | | | | |
| ^c^Wilcoxon rank sum test; Fisher’s exact test; Pearson’s chi-squared test.  ^d^Ever diagnosed for all comorbidities except for depression and/or anxiety, which was diagnosed and/or treated in the past 12 months (2022).  ^e^Mild, moderate, or severe.  BMI, body mass index; COPD, chronic obstructive pulmonary disease; HBVsAg, hepatitis B virus surface antigen; HCV PCR, hepatitis C virus polymerase chain reaction; HRU, healthcare resource utilization; IQR, interquartile range; PTSD, posttraumatic stress disorder; PWH, people with HIV; SES, socioeconomic status; VTE, venous thromboembolic event. | | | | | | | | |
